# Information flow in the rat thalamo-cortical system: spontaneous vs. stimulus-evoked activities

**DOI:** 10.1038/s41598-021-98660-y

**Published:** 2021-09-28

**Authors:** Kotaro Ishizu, Tomoyo I. Shiramatsu, Rie Hitsuyu, Masafumi Oizumi, Naotsugu Tsuchiya, Hirokazu Takahashi

**Affiliations:** 1 Department of Mechano-Informatics, Graduate School of Information Science and Technology, The University of Tokyo, 7-3-1 Hongo, Bunkyo-ku, Tokyo, 113-8656 Japan; 2 Department of General Systems Studies, Graduate School of Arts and Science, The University of Tokyo, 3-8-1 Komaba, Meguro-ku, Tokyo, 153-0041 Japan; 3 School of Psychological Sciences and Turner Institute for Brain and Mental Health, Monash University, Melbourne, VIC Australia; 4 Advanced Telecommunications Research Computational Neuroscience Laboratories, 2-2-2 Hikaridai, Seika-cho, Soraku-gun, Kyoto, 619-0288 Japan; 5 Center for Information and Neural Networks (CiNet), National Institute of Information and Communications Technology (NICT), Suita, Osaka 565-0871 Japan

**Keywords:** Neuroscience, Auditory system, Computational neuroscience, Sensory processing

## Abstract

The interaction between the thalamus and sensory cortex plays critical roles in sensory processing. Previous studies have revealed pathway-specific synaptic properties of thalamo-cortical connections. However, few studies to date have investigated how each pathway routes moment-to-moment information. Here, we simultaneously recorded neural activity in the auditory thalamus (or ventral division of the medial geniculate body; MGv) and primary auditory cortex (A1) with a laminar resolution in anesthetized rats. Transfer entropy (TE) was used as an information theoretic measure to operationalize “information flow”. Our analyses confirmed that communication between the thalamus and cortex was strengthened during presentation of auditory stimuli. In the resting state, thalamo-cortical communications almost disappeared, whereas intracortical communications were strengthened. The predominant source of information was the MGv at the onset of stimulus presentation and layer 5 during spontaneous activity. In turn, MGv was the major recipient of information from layer 6. TE suggested that a small but significant population of MGv-to-A1 pairs was “information-bearing,” whereas A1-to-MGv pairs typically exhibiting small effects played modulatory roles. These results highlight the capability of TE analyses to unlock novel avenues for bridging the gap between well-established anatomical knowledge of canonical microcircuits and physiological correlates via the concept of dynamic information flow.

## Introduction

The interaction between the thalamus and cortex is thought to play critical roles in sensory processing^1,2^. Anatomically, the middle cortical layer is the predominant recipient of thalamocortical projections, whereas the deep cortical layer is the source of cortico-thalamic projections^3–9^. This general structural pattern is observed across different thalamo-cortical systems and mammalian species, and is thus considered a canonical microcircuit in the thalamo-cortical system. These hodological motifs suggest that feedforward pathways originate principally from the supragranular layer (L2/3) and terminate in the granular layer (L4), whereas feedback pathways originate from the infragranular layers (L5/6) and avoid terminating in L4^10–16^. Information flow within these anatomical circuits is thought to be dynamic, with moment-to-moment variation in active pathways^17–21^. For example, communication between the thalamus and cortex is expected to be strengthened during stimulus presentation, whereas communication within the cortex is likely to be strengthened in the resting state in the absence of overt sensory processing. Nevertheless, these differences have yet to be characterized comprehensively in the thalamo-cortical sensory system, especially at the level of neuronal spiking.

Beyond layer-based categorization, further subdivisions of thalamo-cortical pathways have been proposed based on synaptic properties, which may delineate specific roles in information transmission. For example, glutamatergic pathways in the thalamo-cortical system can be classified into either Class 1 or Class 2 (previously termed driver or modulator, respectively). Class 1 inputs express ionotropic glutamate receptors and constitute the main information-bearing pathway, whereas Class 2 projections express metabotropic receptors and modulate the transmission of Class 1 inputs^22–24^. In the auditory system, Class 1 constitutes the main pathway from the ventral division of the medial geniculate body (MGv) to L4–L6 in the primary auditory cortex (A1). Class 2 projections are observed from the MGv to L2/3 and from L5/6 to the MGv^25,26^. Within the cortex, Class 1 and Class 2 are likely intermingled^27–30^. However, this synapse-based pathway characterization has yet to be validated by physiological neural recordings paired with information theoretical analyses, which will enable dynamic and quantitative determination of the nature of information flow.

To characterize the electrophysiological responses in the auditory thalamo-cortical system, we previously designed a microelectrode array that enabled simultaneous neural measurements in the MGv and every layer in A1^31^. In the present study, we used transfer entropy (TE) to characterize pathway-specific information flow in the MGv-A1 system^32–36^. TE is a metric based on information theory that statistically quantifies the directed influence between two sets of time-series data.

Here, we test our hypotheses (i) that TE analyses are able to reveal that information flow during spontaneous activity is distinct to that during stimulus-driven activity, (ii) that TE is able to capture feedforward/feedback flow during stimulus-evoked activity, which is consistent with well-established canonical microcircuits in the thalamo-cortical system, and (iii) that the TEs in MGv-to-A1 direction vary widely because of intermingled projections of Class 1 and Class 2, while A1-to-MGv transmission is commonly characterized as small TEs of Class 2.

## Methods

### Animals

This study was performed in strict accordance with the “Guiding Principles for the Care and Use of Animals in the Field of Physiological Science” published by the Japanese Physiological Society, and the recommendations in the ARRIVE guidelines (https://arriveguidelines.org/). The experimental protocol was approved by the Committee on the Ethics of Animal Experiments at the Research Center for Advanced Science and Technology, University of Tokyo (Permit Number: RAC 130107). All surgeries were performed under isoflurane anesthesia. All efforts were made to minimize animal suffering. Following the experiments, animals were euthanized with an overdose of pentobarbital sodium (160 mg/kg, i. p.).

Four male Wistar rats were used in this study (11–13 weeks old; body weight, 290–330 g). The protocols for animal preparation and neural recordings have been described elsewhere^20,31,37^. Briefly, the rats were anesthetized with isoflurane and air at a concentration of 3% for induction and 1% for maintenance during the surgery and experiments. Animals were held in place with a custom-made head-holding device. Atropine sulfate (0.1 mg/kg) was administered pre- and post-surgery to reduce the viscosity of bronchial secretions. A skin incision was made at the start of surgery under local anesthesia using lidocaine (0.3–0.5 mL). A needle electrode was subcutaneously inserted into the right forepaw and used as the ground. A small craniotomy was performed close to bregma in order to embed a 0.5 mm-thick integrated circuit socket as a reference electrode, with electrical contact to the dura mater. The right temporal muscle, cranium, and dura overlying the auditory cortex were surgically removed. The exposed cortical surface was perfused with saline to prevent desiccation. Cisternal cerebrospinal fluid drainage was performed to minimize cerebral edema. The right eardrum, ipsilateral to the exposed cortex, was ruptured and waxed to ensure unilateral sound inputs from the ear contralateral to the exposed cortex. Respiratory rate, heart rate, and hind-paw withdrawal reflexes were monitored throughout the surgery to ensure maintenance of stable and sufficient anesthesia. For acoustic stimulation, a speaker (Technics EAS-10TH800, Matsushita Electric Industrial Co. Ltd., Japan) was positioned 10 cm from the left ear (contralateral to the exposed cortex). Test stimuli were calibrated at the pinna with a 0.25-inch microphone (4939, Brüel & Kjær, Denmark) and spectrum analyzer (CF-5210, Ono Sokki Co., Ltd., Japan). Stimulus levels were presented in dB SPL (sound pressure level in decibels with respect to 20 μPa).

### Electrophysiology

**Figure 1:**
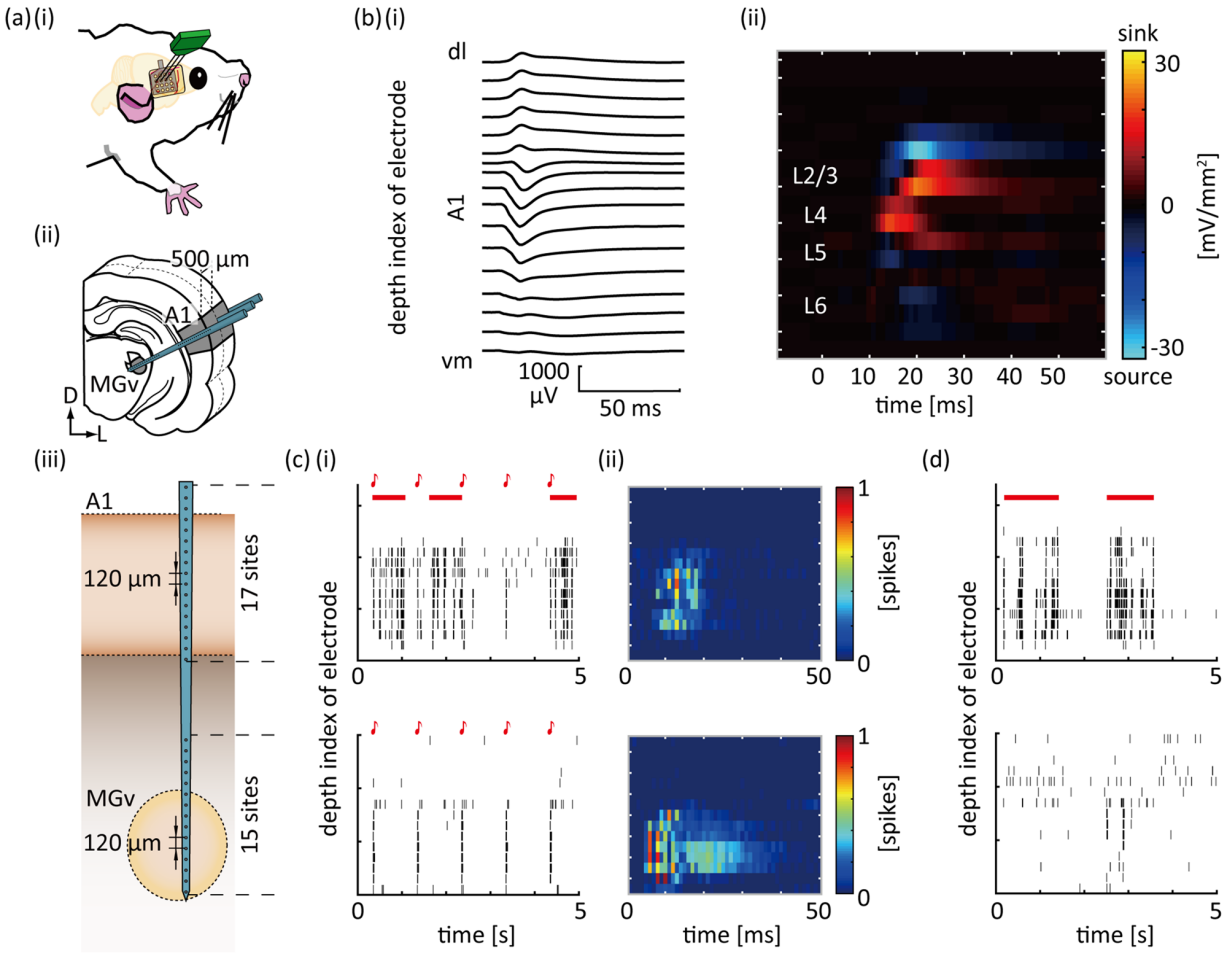
Electrophysiological measurements of the auditory thalamo-cortical system. (**a**) Experimental setup. A custom-designed microelectrode array was used to simultaneously measure multi-unit activity (MUA) and local field potentials (LFPs) from the ventral division of the medial geniculate body (MGv) in the thalamus and primary auditory cortex (A1) of anesthetized rats (**i**). The array was composed of three shanks (6 mm in length) (**ii**), each of which comprised 15 recording sites for the MGv at the distal edge and 17 recording sites for A1 on the proximal side (**iii**). (**b**) Identification of cortical layers. For LFPs in response to a click sound (**i**), current source density analysis (CSD) was performed (**ii**). (**c**) Click-evoked MUA in the MGv (bottom) and A1 (top). Raster plots for 5 s (**i**) and discharge rates within a given bin (**ii**) at 32 recording sites in a representative shank are depicted. The longitudinal axis corresponding to the depth of recording sites as depicted in inset (**a-iii**). (**d**) Spontaneous MUA in the MGv (bottom) and A1 (top). Data in (**c**) and (**d**) were obtained from the same rat. Red lines indicated burst periods in (**c**-**i**) and (**d**-**i**).

We used a surface microelectrode array and depth electrode array (NeuroNexus, Ann Arbor, MI, USA) to simultaneously measure neural activity in the auditory cortex and thalamus, as previously described^28^ (Fig. 1a). The surface microelectrode array comprising a 10 × 7 grid within 4 × 3 mm^2^ mapped local field potentials (LFPs) in the right temporal cortex to identify the location of the primary auditory cortex (A1)^37^. The depth microelectrode array was then inserted perpendicular to the cortical surface in A1. The array comprised three shanks (6 mm in length), each of which constituted 15 distal recording sites for MGv and 17 proximal sites for A1. The array simultaneously measured multi-unit activity (MUA) and LFPs from the MGv and A1. The diameter of recording sites was 30 μm. The center-to-center inter-electrode distance was 120 μm. The most distal site was placed 100 μm from the tip of the shank, and the distance between the most proximal site in the MGv and the most distal site in A1 was 1200 μm. Each electrode was composed of iridium oxide and coated with platinum black.

Neural signals were amplified with a gain of 1000 (Cerebus Data Acquisition System; Cyberkinetics Inc. Salt Lake City, UT, USA) software. The digital filter bandpass was 0.3–500 Hz for LFP and 250–7500 Hz for MUA. The sampling rates for LFPs and MUA were 1000 Hz and 30 kHz, respectively. Multi-unit spikes were detected online from MUA by threshold-crossing (− 5.65 times root mean square of MUA).

Spontaneous activity was first characterized as MUA in a silent environment for 5 min. Auditory-evoked activity was then characterized in response to clicks and tone bursts. Clicks were presented at a rate of 1 Hz. Tone bursts were used to characterize the characteristic frequency (CF) at each recording site. CF was determined as the frequency at which test tones evoked MUA with the lowest intensity or the largest response at 20 dB SPL (the minimum intensity used in this study). Test frequencies ranged from 1.6 to 64 kHz with an increment of 1/3 octaves and intensities from 20 to 80 dB SPL with an increment of 10 dB. Each test tone was repeated 20 times in a pseudorandom order with an inter-tone interval of 600 ms. Recording sites at which CF was identified were defined as either MGv or A1, whereas those at which CF was not identified were excluded from further analyses.

For the grand average of 240-trial click-evoked LFPs from the depth array, one-dimensional current source density (CSD) analysis (Fig. 1b) was conducted, as described previously^28,38,39^. Briefly, twice the potential at a given depth (V_0_) was subtracted from the sum of the potentials at the upper and lower adjacent sites of a given depth (V_u_ and V_l_), and then divided by the square of the distance (Δ*x*) between the recording sites (120 μm):

$$\left( {{\text{V}}_{{\text{u}}} + {\text{ V}}_{{\text{l}}} - {\text{ 2V}}_{0} } \right)/\Delta x^{{2}} .$$

Each layer was defined based on the CSD results as follows: L4 was first defined as the site with the earliest sink and adjacent sites as sinks and no source. L2/3 was defined as sites above L4 with sinks, followed by short sources. L5 was defined as two successive sites with sources below L4. Weak sinks were identified in deeper sites, of which the second deeper site was defined as L6.

### Transfer entropy

**Figure 2:**
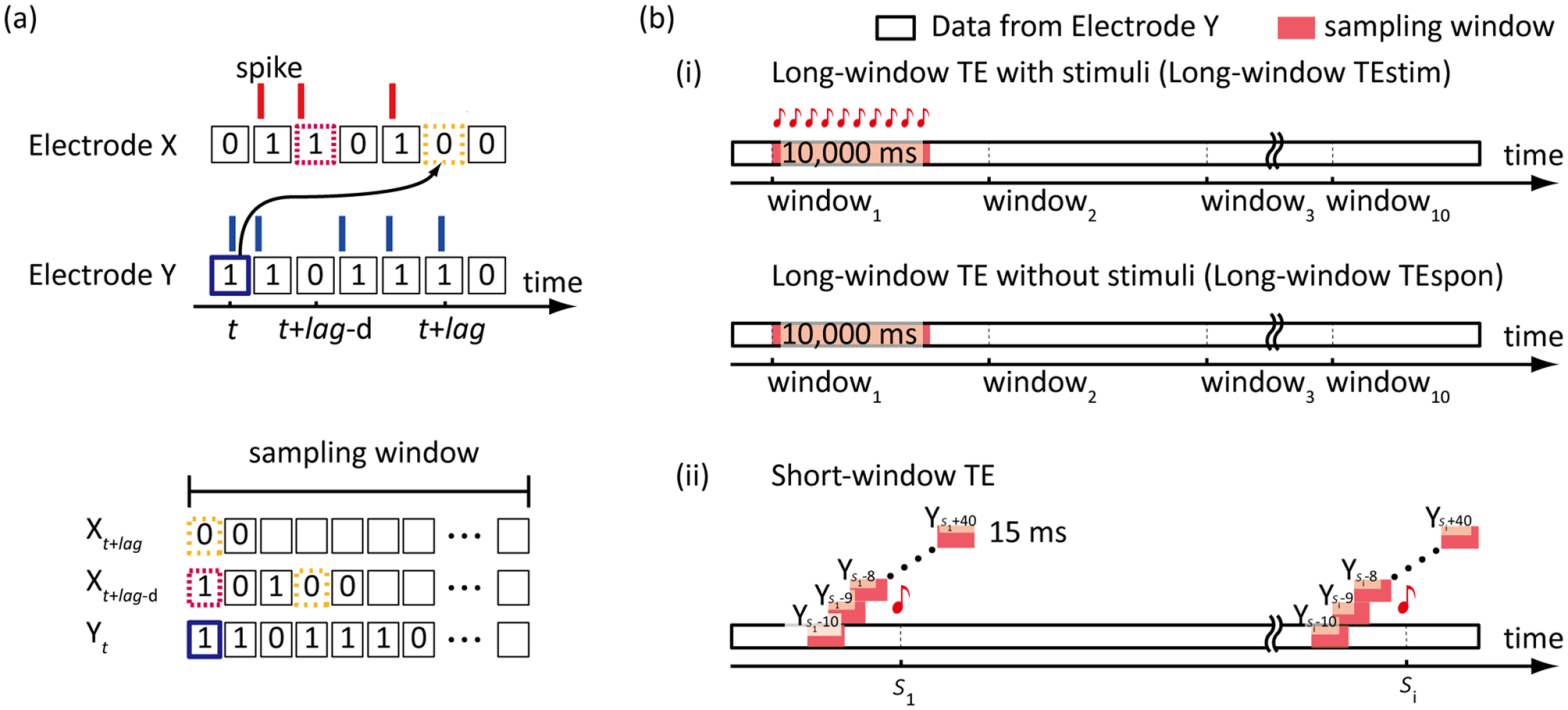
Estimation of transfer entropy from spike train data. (**a**) Schematic of transfer entropy (TE) from electrodes Y to X. Binned spike train data sets were generated from MUA data with 1-ms bins. The joint probability $p\left({X}_{t+lag},{X}_{t+lag-d},{Y}_{t}\right)$ was estimated within a certain sampling window. See text for details. (**b**) Sampling window for TE analyses. (**i**) Long window of 10 s. TE was computed for neural activity with and without stimulus presentation (long-window TEstim and long-window TEspon, respectively). (**ii**) Short window of 15 ms. The time course of short-window TE was examined using moving window analyses.

TEs of either thalamo-cortical, intracortical, or cortico-thalamic projections were derived from MUA data of either spontaneous activity or click-evoked activity in a pairwise manner. TE was estimated from MUA data binarized with a bin of 1 ms (Fig. 2a). Bins with spikes were labeled as 1; those without spikes were labeled as 0. None of the bins contained two or more spikes. The TE of Y to X or ${TE}_{Y\to X}$ was defined as follows:

1 $${TE}_{Y\to X}=H\left({X}_{future}|{X}_{past}\right)-H\left({X}_{future}|{X}_{past},{Y}_{past}\right)$$

where *H*(A|B) represents the conditional entropy in information theory, which indicates the unpredictability of A when information on B is known. ${TE}_{Y\to X}$ estimates how spikes at electrode Y (${Y}_{past}$) improve the prediction of spikes at electrode X (${X}_{future}$), beyond the prediction based on past data of X (${X}_{past}$). Here, ${TE}_{Y\to X}$ was calculated as follows:

2 $${TE}_{Y\to X}(t, lag)=\sum_{\begin{array}{c}{X}_{t+lag}\\ {X}_{\begin{array}{c}t+lag-d\\ {Y}_{t}\end{array}}\end{array}}p\left({X}_{t+lag},{X}_{t+lag-d},{Y}_{t}\right) {log}_{2}\frac{p\left({{X}_{t+lag}|X}_{t+lag-d},{ Y}_{t}\right)}{p\left({X}_{t+lag}|{X}_{t+lag-d}\right)},$$

where *t*, *lag*, and *d* represent the time, transfer lag, and delay, respectively, between the future and past. ${Y}_{t}$ represents the past state of electrode Y (${Y}_{past}$). ${X}_{t+lag}$ and ${X}_{t+lag-d}$ represent the future and past states, respectively, of electrode X (${X}_{future}$ and ${X}_{past}$). The past data of X were obtained from *d* bins before a given time point of (*t* + *lag*), which were optimized as follows, assuming that X_*t*_ depends predominantly on past X_*t-d*_:

3 $$d = argmin H\left( {X_{t} |X_{t - d} } \right) \ldots$$

According to Eq. (1), we quantified ${TE}_{Y\to X}$ for given electrode pairs with either a short window (15 ms) or long window (10 s) (Fig. 2b).

(i) *Long-window TE with and without stimuli (long-window TEstim and long-window TEspon, respectively)*

Long-window TE was derived using 10-s windows to assess if information transmission differed depending on the state of the thalamo-cortical system (i.e., during sensory processing vs. resting state). Long-window TEstim was derived from MUA over a continuous period of 240 s, during which clicks were presented every second. Long-window TEspon was derived from a separate 240-s time period of data during which no stimulus was delivered. Ten sets of 10-s $p\left({X}_{t+lag},{X}_{t+lag-d},{Y}_{t}\right)$ and spike trains were randomly selected to derive the joint probability, $p\left({X}_{t+lag},{X}_{t+lag-d},{Y}_{t}\right)$. Based on Eq. (1), 10 sets of TE were then estimated in the transfer lag ranging between 1 and 30 ms. Long-window TEs were ultimately defined as the median across 10 sets for each *lag*.

(ii) *Short-window TE*

Short-window TE was computed using 15-ms windows to characterize information transmission in the thalamo-cortical system during the time window surrounding stimulus onset. The time course of information transmission for short-window TE was investigated using moving window analysis.

For trial *i* (= 1, …, 240), in response to a click delivered at time *s*_*i*_, spike trains within 15-ms post-stimulus latency were used to derive short-window TE. Based on short-window TE at stimulus onset (short-window TEonset), we first identified significant information transmission and the optimal transfer lag, *Lag*_*opt*_, for a given electrode pair. For $\left[{s}_{i}+1, {s}_{i}+15\right] := \left\{{s}_{i}+1\le t\le {s}_{i}+15\right\}$, the joint probability, $p\left({X}_{t+lag},{X}_{t+lag-d},{Y}_{t}\right),$ was obtained to derive ${TE}_{Y\to X}$ at a given *lag* (= 1, …, 30) according to Eq. (2). Short-window TEonset was ultimately defined as the median across 240 trials for each *lag*. The optimal transfer lag, *Lag*_*opt*_, was determined as the transfer lag that maximized the short-window TEonset.

We next characterized the time-course of short-window TE, i.e., how TE evolved over time in the thalamo-cortical system during the time window surrounding stimulus onset. We computed the short-window TE for $\left[T-\frac{15+{Lag}_{opt}}{2}, T+\frac{15+{Lag}_{opt}}{2}\right] := \left\{T-\frac{15+{Lag}_{opt}}{2}\le t\le T+\frac{15+{Lag}_{opt}}{2}\right\}$, where *T* ranged from *s*_*i*_ -10 to *s*_*i*_ + 40 and the *lag* was the optimal value in the short-window TEonset. When *t* was not an integer, *t* was rounded off to the nearest integer. The time course of short-window TE was ultimately defined as the median across 240 trials for each *T*. The earliest *T* when TE > 0 after bias correction (see the next section) was defined as the onset latency of information transmission.

### Statistical analyses for identification of significant information transfer

To identify electrode pairs with significant information transfer, we compared the above TEs derived from experimental data with those derived from shuffled data (TE_shuffled_). To generate the shuffled data, we randomly shuffled the inter-spike intervals (ISIs) of X_t_ and Y_t_ without changing the ISI distribution. Shuffling disrupted the temporal structure underlying functional connectivity between X_t_ and Y_t_.

**Figure 3:**
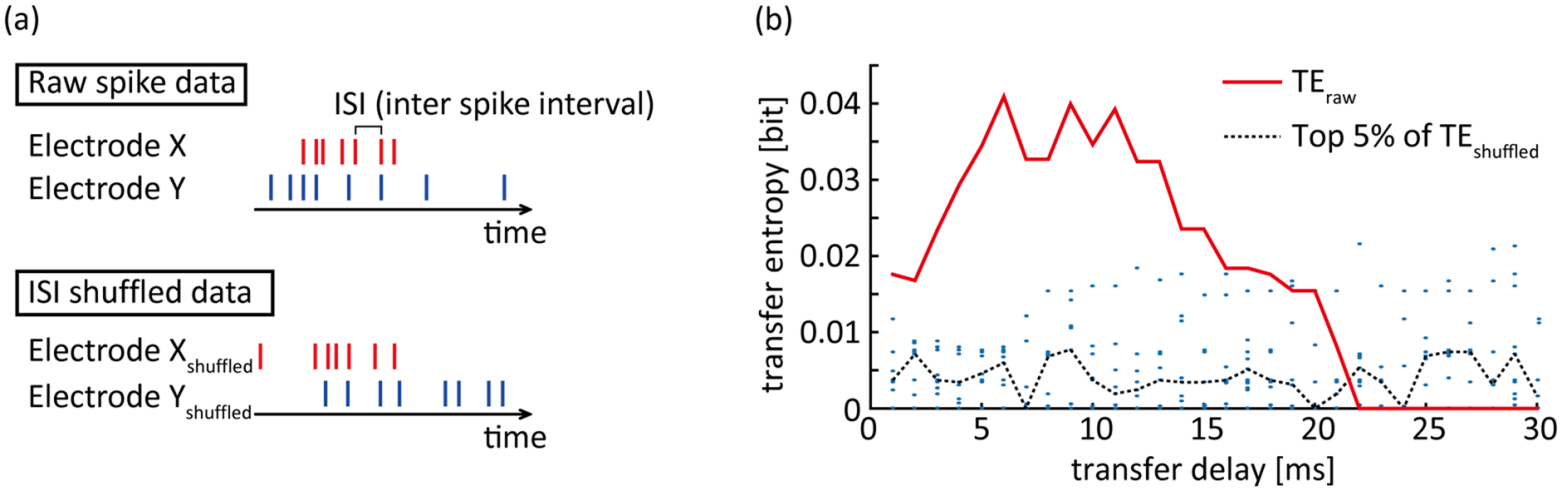
Identification of significant information transfer. We shuffled the raw spike data to generate 100 datasets without changing inter-spike interval (ISI) distribution (**a**). Transfer entropy (TE) values were derived from either raw spike data (red line; TE_raw_) or shuffled data (blue dots; TE_shuffled_) at every transfer delay (**b**). Significant information transfer was defined as TE_raw_ exceeding the top 5% of 100 TE_shuffled_ (black dotted lines). An electrode pair was defined as having functional connectivity if significant information transfer was observed within a time window of 5 ms or more.

To assess statistical significance of information transfer, we estimated *p*-values as the rank order of empirically identified TE values among the null distributions arising from 100 TE_shuffled_. For example, if the empirical TE was larger than the top 5% of 100 sets of TE_shuffled_, we regarded the *p*-value to be less than 0.05^38^. We corrected for multiple comparisons across transfer lags (1–30 ms) using the false discovery rate (FDR) method^39^. Further, we defined a pair of functionally connected electrodes as those with significant information transfer within a time window of 5 ms or more (Fig. 3).

When quantifying the amount of information transfer, we considered the degree of positive bias caused by a limited amount of sample data. Theoretically, TE_shuffled_ must become 0 because shuffling should disrupt any causality between X and Y. However, the actual TE_shuffled_ was larger than 0 due to biases, which were removed by subtracting the median TE_shuffled_ from the TE. When TE was smaller than TE_shuffled_, no information transfer was assumed (i.e., TE = 0).

### Normalized TE (nTE)

Mean firing rates of evoked activity was substantially higher than those of spontaneous activity (Fig. 1c,d). To eliminate the bias due to differences in mean firing rate, we introduced the nTE. This normalization was necessary when comparing TEs derived from evoked and spontaneous states with different probability densities as follows:

$${nTE}_{Y\to X}=\frac{H\left({X}_{future}|{X}_{past}\right)-H\left({X}_{future}|{X}_{past},{Y}_{past}\right)}{H\left({X}_{future}|{X}_{past}\right)}= \frac{{TE}_{Y\to X}}{H\left({X}_{t+lag}|{X}_{t+lag-d}\right)}$$

Practically, the bias of nTE was corrected as

4 $$n {TE}_{Y\to X}= \frac{{TE}_{Y\to X}-{TE}_{Y\to X}^{shuffled}}{H\left({X}_{t+lag}|{X}_{t+lag-d}\right)} \in [\mathrm{0,1}]\dots$$

### Information transmission in a given pathway

We characterized the information transmission in each pathway as the average of the peaks of nTE among pairs with significant information transfer:

5 $$Average\,of\,nTE\,peaks = \frac{1}{n}\sum (peak\,of\,nTE)\times \frac{n}{{N}_{pathway}}\dots$$

where *n* is the number of pairs with significant information transfer, and ${N}_{pathway}$ is the number of possible pairs of electrodes.

### Role of a given region in information transmission

Based on the average of the nTE peaks defined above, we quantified whether each region (X) served as either a receiver (${R}_{X}$) or a sender (${S}_{X}$). The metrics ${S}_{X}$ and ${R}_{X}$ were defined as the summation of the average of the nTE peaks as follows:

6 $${S}_{X}={\sum }_{i}average\,of\,nTE\,peaks\left(X\to {region}_{i}\right)\dots$$

7 $${R}_{X}={\sum }_{i}average\,of\,nTE\,peaks\,\left({region}_{i}\to X\right)\dots$$

${region}_{i}$: one of (MGv, L2/3, L4, L5, and L6) with the exception of $X$

where *the average of nTE peaks(pathway)* is the average of nTE peaks in a given pathway, as defined in Eq. (5). We then characterized each region *X* using the *SR ratio*:

8 $$SR\,ratio=\frac{{S}_{X}-{R}_{X}}{{S}_{X}+{R}_{X}} \in [-1, 1]\dots$$

A positive *SR ratio* indicated that region *X* served as a sender, whereas a negative *SR ratio* indicated that region *X* served as a receiver.

### Burst detection

Under isoflurane anesthesia, cortical LFPs exhibited an alternating pattern of high amplitude bursts and suppressed activity, which might influence the information flow and transmission in the thalamo-cortical system. The burst activities were easily detectable because they simultaneously appeared in all recording sites over the auditory cortex^21,40^. For the burst detection, the variances of LFP over time per electrode were measured with every 100-ms window at each time point. If the variance exceeded a threshold over 100 ms in > 25 (out of 96) electrodes, we regarded the analyzed period as burst. The burst threshold was identified heuristically.

## Results

In the four rats tested, 96 sites in the MGv and 138 sites in A1 exhibited tone-evoked MUA, which exhibited definable auditory responses and a CF. Among these sites, we simultaneously measured click-evoked and spontaneous MUA in the MGv and A1 (Fig. 1c,d). Under isoflurane anesthesia, burst activities were often observed as barrages of MUAs in A1 (red lines in Fig. 1c,d), which might influence the information flow. To address this concern, we first measured the burst period in our data. As a result, burst periods did not depend on stimulus presentation significantly (mean ± SD: 25.3 ± 5.4 s/min in spontaneous activities vs. 30.2 ± 5.8 s/min during stimulus presentation; t-test, *P* = 0.2), and hence, bursts were likely to have little effects to characterize the difference between TEspon and TEstim.

We derived long-window TE and short-window TE between all possible pairs among available sites. Significant information transfer was identified in 11,483 pairs (98% of all possible pairs) in long-window TEstim, 3964 pairs (36%) in long-window TEspon, and 5246 pairs (45%) in short-window TEonset. These significant pairs were further characterized as follows. Among these pairs, 2430, 107, and 1578 pairs (83%, 4%, and 54% of pairs, respectively) transmitted information from the MGv to A1; 2005, 108, and 743 pairs (69%, 4%, and 25% of pairs, respectively) transmitted information from A1 to MGv; and 2671, 2548, and 1405 pairs (75%, 71%, and 40% of pairs, respectively) transmitted information from A1 to A1 in long-window TEstim, long-window TEspon, and short-window TEonset, respectively. Supplementary Fig. S1 summarizes the distribution of CF at test sites in A1 and MGv, and the difference of CF (delta CF) among pairs with significant TE. Because each shank of the microelectrode array penetrated a single tonotopic column in A1, the CFs in A1 were more narrowly distributed than those in MGv. As the figure shows, the majority of delta CF was confined within ± 1 octave. To test if pairs with significant TE have the matched CF, we performed the following. Using bootstrap analysis, we constructed surrogate delta CF distributions by randomly sampling MGv and A1 sites by resampling from the real distribution. We found this distribution did not differ from the real distribution. This suggests that this relatively narrow range of delta CF was caused by the sampling bias of CF at test sites. In sum, we interpret this to mean that significant TE pairs did not have matched CF more often than unmatched CF, at least in our data of click-evoked MUAs.

### Long-window TE

**Figure 4:**
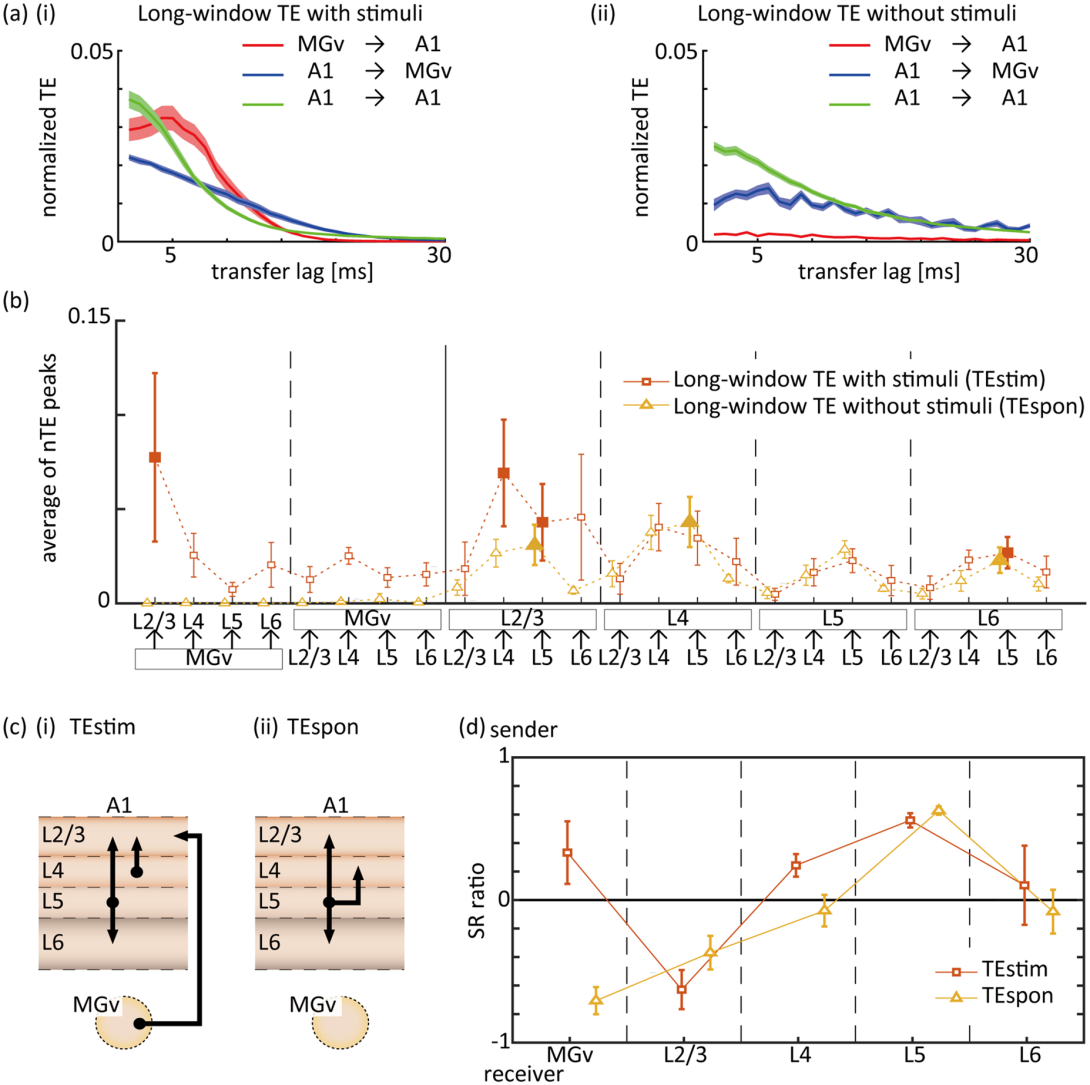
Long-window TE differentiates information flow with and without stimulus presentation. (**a**) Normalized TE (nTE) as a function of transfer lag: (**i**) nTE during stimulus presentation (long-window TEstim) and (**ii**) nTE during spontaneous activity (long-window TEspon). Average and standard error across subjects are presented. Colors indicate pathways: red, MGv-to-A1; blue, A1-to-MGv; green, A1-to-A1 pathways. (**b**) Information flow in a given pathway. Recording sites in A1 were classified into Layers 2/3, 4, 5, and 6. TEstim and TEspon were characterized in four thalamo-cortical pathways, four cortico-thalamic pathways, and 16 intracortical pathways between cortical nodes (L2/3, L4, L5, and L6) and nodes in the MGv. Information flow in each pathway was quantified as the average of nTE peaks (see text for details). The filled symbols indicate that information flow was significantly larger than that in the opposite pathway (one-sided Student’s t-test, *P* < 0.05). (**c**) Schematic diagram of information flow. Significant directional influences in (**b**) are depicted. (**d**) Sender/receiver (SR) ratio at each region. SR ratios were derived for TEstim and TEspon. Regions with a positive SR ratio served as senders, whereas those with a negative ratio served as a receiver.

For long-window TE, we compared information flow with and without stimulus inputs. The nTE of (i) long-window TEstim and (ii) long-window TEspon as a function of transfer lag is presented in Fig. 4a. TEstim decayed abruptly at a transfer lag of approximately 15 ms in the MGv-to-A1 (red) direction, whereas it decayed smoothly in the A1-to-MGv (blue) direction (Fig. 4a-i). TEstim in the feedforward pathway from MGv to A1 and intracortical pathway was larger than that in the feedback pathway from A1 to MGv. TEspon (Fig. 4a-ii) indicated that the forward transmission of information from MGv to A1 almost disappeared during spontaneous activity compared to TEstim. These results support the notion that stimulus-driven information flow is distinct to spontaneous information flow and that each pathway possesses different information flow properties.

We further subdivided the cortical recording sites into four different layers (L2/3, L4, L5, and L6) according to the CSD analysis (Fig. 1b) and characterized information flow as the averages of nTE peaks in 24 pathways (Fig. 4b), as defined in Eq. (5). To evaluate the consistency of this measure across subjects, we compared information flow patterns of 24 pathways across subjects and quantified the correlation coefficients of the patterns between subjects in a pairwise manner. The correlation coefficients across subjects for TEstim and TEspon were 0.665 $\pm$ 0.185 and 0.954 $\pm$ 0.013 (average $\pm$ SD), respectively (t-test: TEstim, *P* < 0.05 in 5 out of 6 pairs of test rats; TEspon, *P* < 10^−11^ for all pairs; see Supplementary Fig. S2a and S2b). The moderately high correlation coefficient in the presence of stimuli (0.665) and high correlation coefficient during spontaneous activity (0.954) verified the ability of our analyses to capture general patterns of information flow.

We next investigated the directionality of information flow. Based on TEstim and TEspon, the filled symbols in Fig. 4c-i,c-ii indicate that information flow was significantly larger than that in the opposite direction (one-sided Student’s t-test; *P* < 0.05). These pathways are highlighted in the schematic diagram of the thalamocortical system in Fig. 4c. Neural activity originating from L5 spread to other cortical layers and terminated in L2/3 in both TEstim and TEspon. In the presence of stimulation, additional activity originated from the MGv and L4. These trends of information flow were supported by data presented in Fig. 4d, which characterized the role of each region as either a sender or receiver based on the SR ratio as defined by Eq. (8).

### Short-window TE

**Figure 5:**
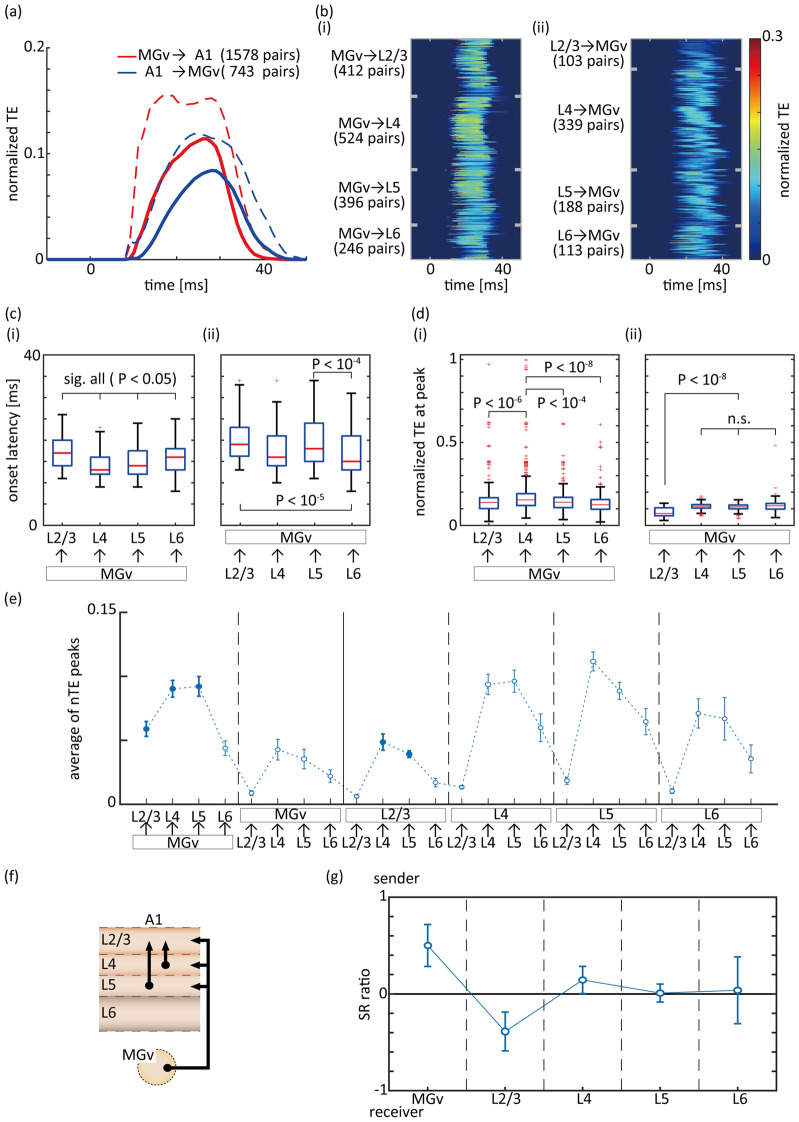
Short-window TE to characterize feedforward and feedback information transmission. (**a**) The average time-course of short-window TE between MGv and A1: red, feedforward thalamo-cortical pathway (i.e., from MGv to A1); blue, feedback cortico-thalamic pathway (i.e., from A1 to MGv). The average and standard deviation (solid and dotted lines) are provided for available pairs. (**b**) Time-course of TE for individual test pairs. (**i**) Feedforward pathway. (**ii**) Feedback pathway. Cortical nodes were classified into Layers 2/3, 4, 5, and 6 (L2/3, L4, L5, and L6). (**c**) Onset latency of nTE transfer. (**d**) nTE at the peak in the time-course analyses. For each box, the central mark indicates the median (across all available/significant pairs and animals), and edges indicate the 25th and 75th percentiles. The whiskers extend to the most extreme data points that were not considered outliers, which exceeded the 75th percentile or were less than the 25th percentile by 1.5 times the inter-quartile range. Large outliers, i.e., nodes that exerted extremely large influence, were more frequently observed in the feedforward direction than in the feedback direction. (**e**) Information transmission in a given pathway. (**f**) Schematic diagram of information transmission. (**g**) Sender/receiver (SR) ratio at each region. For (**e**–**g**), see conventions in Fig. 4c–e.

Based on the above analysis, we conjectured that sender/receiver characteristics may change as a function of time during the temporal window surrounding stimulus onset, and that feedforward and feedback information transmission may be temporally segregated. For each significant electrode pair in the short-window TEonset, the time course of short-window TE was derived using moving window analyses (Fig. 2b). We first compared information transmission in the feedforward (i.e., 1578 pairs from MGv to A1; 54% of all possible pairs) and feedback direction (i.e., 743 pairs from A1 and MGv; 25% of all possible pairs). For both the average of all test pairs (Fig. 5a) and individual test pairs (Fig. 5b), we observed that feedforward information transmission was larger in amount and earlier in latency compared to feedback information transmission.

To test whether feedforward pathways (Fig. 5b-i) were activated earlier than feedback pathways (Fig. 5b-ii), we quantified the onset latency of (i) feedforward and (ii) feedback information transmission in each layer-specific pathway (Fig. 5c). L4 received feedforward information with the earliest onset from MGv, whereas L2/3 received information with the latest onset (Fig. 5c-i; Kruskal–Wallis followed by Tukey–Kramer test, *P* < 0.05 for all pairs). In contrast, feedback information transmission from A1 to MGv was initiated in L6 (Fig. 5c-ii). These properties of feedforward information transmission were consistent with previous physiological findings^10–16^, underscoring the major benefits of the estimates of feedback transmission in our analyses.

To dissect the differences between feedforward and feedback pathways in more detail, we quantified feedforward and feedback information transmission in each layer-specific pathway (Fig. 5d). L4 received the largest influence from MGv (Fig. 5d-i; Kruskal–Wallis followed by Tukey–Kramer test, L4 vs. L2/3 (*P* < 10^−6^), L5 (*P* < 10^−4^), and L6 (*P* < 10^−8^)). In contrast, L2/3 exerted the least influence on MGv in the feedback pathway (*P* < 10^−8^) (Fig. 5d-ii). These results are consistent with well-established anatomical projections^10–16^. Notably, information transmission in the feedforward direction contained several outliers which exerted extremely large influences. In contrast, information transmission in the feedback direction was more normally distributed. This observation is consistent with the notion that a subpopulation of projections in the feedforward pathway is information-bearing, i.e., Class 1 projection, and that feedback pathways play a modulatory role through Class 2 projection^22–30^.

Similar to the analysis for long-window TE, we characterized information transmission based on short-window TE in 24 pathways (Fig. 5e). The correlation coefficient of information transmission patterns of the 24 pathways across subjects was 0.629 ± 0.148 (*P* < 0.05 for all test pairs; see Supplementary Fig. S2c). The filled symbols in Fig. 5e indicate that information transmission was significantly larger than that in the opposite direction (one-sided Student’s t-test; *P* < 0.05). These pathways are highlighted in the schematic diagram of the thalamocortical system in Fig. 5f. Neural activity originated from MGv, L4, and L5, and terminated in L2/3. As depicted in Fig. 5g, MGv served as a sender and L2/3 as a receiver, whereas L4 and L5 served as relay stations with inward and outward transmission of similar magnitude. The difference between long-window TEstim (Fig. 4c-i) and short-window TE (Fig. 5f), i.e., L5 not always serving as a sender, indicated that the MGv was the origin of stimulus-driven information transmission in the thalamo-cortical system.

Lastly, Supplementary Fig. S3 characterized how the information transmission between MGv and A1 depended on the tonotopic axis, i.e., whether CF of a test pair matched (delta CF ≤ 1/3 oct) or unmatched (delta CF > 1). In the feedforward pathways, i.e., from MGv to A1, the amount of transmission did not depend on the tonotopic axis (Fig. S3a). In terms of onset latency, L4 and L5 in A1 tended to receive information from MGv earlier than other layers (Fig. S3b; *P* < 0.05), consistent with the direct tonotopic projection from MGv to L4 and L5^31^. In the feedback pathways, L6 tended to send more information to MGv in pairs with unmatched CFs than in those with matched CFs (Fig. S3c; *P* < 0.05) although no difference was found in latency (Fig. S3d). Such feedback connectivity might play crucial roles in regulating gain and selectivity along the tonotopic axes in the thalamocortical system^41^.

## Discussion

In this study, we performed TE analysis to characterize information flow in the thalamo-cortical pathway between the MGv and A1 in anesthetized rats. We simultaneously recorded MUA in both the MGv and A1 (Fig. 1), and estimated TE using two different sampling windows (Fig. 2). We employed long-window TE to compare information flow with and without stimulus presentation, and short-window TE to scrutinize feedforward and feedback transmission around the time of stimulus onset. TE analyses were applicable to MUA data to characterize such bidirectional information flows, while traditional cross-correlation approach^42,43^ can be in principle applied to MUA data. However, in such a case, we cannot unambiguously interpret which MUA caused the other (as MUA represents spikes of many neurons). Further, traditional cross correlation applied to single-unit activities is also difficult to interpret when the firing rate is very low. Our analyses were consistent with well-established neuroanatomical literature and demonstrated that information flow was dynamic, with moment-to-moment variability in active pathways depending on the mode of stimulus processing.

Long-window TEspon indicated that thalamo-cortical information flow almost disappeared and intracortical communication became dominant during spontaneous activity. Short-window TE revealed that the MGv acted as a sender to L4 and L5 in response to a click stimulus. The time-course of short-window TE demonstrated that feedforward thalamo-cortical information flow preceded feedback cortico-thalamic information flow. L4 received the greatest influence and earliest latency from the MGv in the feedforward direction. These results are in accordance with well-characterized anatomical structures and canonical microcircuits in the thalamo-cortical system, thus confirming the validity of our analyses^3–15^. However, this structure of information flow was not revealed by long-window TEstim, in which L4 and L5 were not predominant receivers from MGv, and instead, L2/3 was the predominant receiver. This discrepancy between short-window and long-window TE was caused by the temporal resolution of the analyses. The 10-s window of long-window TEstim was able to capture some thalamo-cortical information flows, i.e., MGv-to-L2/3, but was too long to reveal the precise pathways, i.e., MGv-to-L4/5, which were active only within 50 ms following the stimulus onset.

Our analyses revealed several differences in aspects of information flow between the MGv and A1. First, the information transfer window was approximately 15 ms for MGv-to-A1 transmission, which was narrower than that in the A1-to-MGv direction (Fig. 4a-i). This order of transfer lags for information transmission to A1 is consistent with previous findings^44^. Such transfer lags are substantially longer than the cortical synaptic delay of 2 ms^43,45^ and are therefore likely generated within abundant recurrent connections in A1, but not in MGv^11–13,46,47^. Further, the order of the time window for integration is reminiscent of a cycle of gamma-band oscillations, which are generated via the interaction between pyramidal and inhibitory interneurons^48–50^ and subserve information integration^51–55^. Second, the influence of A1-to-MGv nodes was typically small (Fig. 5d-ii), whereas the influence of MGv-to-A1 nodes varied considerably (Fig. 5d-i). Furthermore, the outliers in Fig. 5d-i imply that a small proportion of MGv-to-A1 information transmission was significantly higher than the average value, indicative of high efficiency in driving post-synaptic neurons. These information-bearing nodes are likely to be classified as Class 1 projections, which express glutamatergic ionotropic receptors. In contrast, other nodes may be classified as Class 2 projections, which express metabotropic receptors^22–30^. The MGv may comprise more Class 1 pathways compared to A1, enabling transfer of external stimulus information (outliers in Fig. 5d-i vs. d-ii).

Our analyses demonstrated that communication between the thalamus and cortex is strengthened during stimulus presentation, whereas communication within the cortex is strengthened during spontaneous activity. Furthermore, differences and similarities between long-window TEstim and long-window TEspon provide critical insight into cortical computational processes. For example, our results in Fig. 4 are consistent with past reports in that the major source of information flow during spontaneous activity likely originated from L5^17,56^. L5 is more likely to serve as the source of spontaneous activity in A1 because L5 exhibits more depolarized membrane potentials and higher firing rates compared to other layers^47,57,58^ with less inhibition^59,60^. During both spontaneous and evoked activity, L2/3 constituted more pathways for information inflow than for information outflow. These patterns of information flow suggest that L2/3 has a higher-dimensional space of representation compared to L4, corroborating the conceptual framework of sparse coding formation in L2/3 from high activity in L4^17,47,57,61,62^.

Our pairwise estimation of information flow has several limitations, which may complicate the interpretation of our results. Although monosynaptic connections in L5 were previously identified in 0.25% of test pairs^43^, significant information flow was observed for 25–49% of possible test pairs in our analyses, suggesting that TE based on MUA was a measure of polysynaptic connections, rather than monosynaptic connectivity. Furthermore, false-positive TE may have been obtained for a pair of nodes which both receive projections from a common origin^33,34,63^. For example, a proportion of information flow between L4 and L5 may have been false positives, because both L4 and L5 receive dense projections from the MGv^3,4,7,8,11–13,15,26,64–66^. This false positive information flow may be more frequently observed in the L4-to-L5 direction than in the opposite direction because click-evoked responses occur earlier in L4 than in L5. To overcome these limitations, conditional mutual information methods such as momentary TE^67^ should be employed to estimate direct causality by conditioning out the effects of possible common drivers. Other alternatives to reduce the effects of common drivers exist^68–71^. Nevertheless, these techniques share the issue of estimation of neural interactions when the number of nodes in the analysis is large. Given that we have 96 electrode sites, there are currently no known techniques to address the problem of combinatorial explosion. Therefore, we believe that the pairwise TE is a well-balanced solution at present to give reasonable validation from the anatomical and physiological viewpoints.

In conclusion, we simultaneously measured MUA in the MGv and A1 in rats and harnessed TE analyses to characterize information flow in the auditory thalamo-cortical system. Long-window TE revealed that communication between MGv and A1 was strengthened during stimulus presentation, whereas thalamo-cortical communications almost disappeared and intracortical communications were strengthened during spontaneous activity. Short-window TE indicated that feedforward (thalamo-cortical) information transmission was followed by feedback (cortico-thalamic) transmission at stimulus onset, and L4 exerted the largest influence with the earliest latency from the MGv in the feedforward direction, corroborating anatomical reports on thalamo-cortical projections. Furthermore, consistent with the notion of Class 1 and Class 2 synaptic properties, a small but significant population of MGv-to-A1 pairs was information-bearing, whereas A1-to-MGv pairs that typically exhibited a small influence were likely to play modulatory roles. Our results highlight the capability of TE analyses to unlock novel avenues for bridging the gap between well-established anatomical knowledge of canonical microcircuits and physiological findings via the concept of dynamic information flow.

## Supplementary Information

Supplementary Information.

## Data Availability

The datasets generated during and/or analysed during the current study are available from the corresponding author on reasonable request.
