# Supplementary material for: Information flow in the rat thalamo-cortical system: spontaneous vs. stimulus-evoked activities
Source: Sci Rep. 2021 Sep 28;11:19252. doi: 10.1038/s41598-021-98660-y (PMC8479136; doi:10.1038/s41598-021-98660-y)
Supplement: Supplementary file 1 — Supplementary Information. [file 41598_2021_98660_MOESM1_ESM.docx]

Supplementary Information

**Information flow in the rat thalamo-cortical system: spontaneous vs. stimulus-evoked activities**

Kotaro Ishizu^1^, Tomoyo I. Shiramatsu^1^, Rie Hitsuyu^1^, Masafumi Oizumi^2^, Naotsugu Tsuchiya^3,4,5^, and Hirokazu Takahashi^1*^

^1^ Department of Mechano-informatics, Graduate School of Information Science and Technology, The University of Tokyo, Japan.

^2^ Department of General Systems Studies, Graduate School of Arts and Science, The University of Tokyo, Japan.

^3^ School of Psychological Sciences and Turner Institute for Brain and Mental Health, Monash University, Melbourne, Victoria, Australia

^4^ Advanced Telecommunications Research Computational Neuroscience Laboratories, 2-2-2 Hikaridai, Seika-cho, Soraku-gun, Kyoto 619-0288, Japan.

^5^ Center for Information and Neural Networks (CiNet), National Institute of Information and Communications Technology (NICT), Suita, Osaka 565-0871, Japan

*Corresponding author:

Hirokazu Takahashi

Associate Professor

Department of Mechano-informatics

Graduate School of Information Science and Technology

The University of Tokyo

7-3-1 Hongo, Bunkyo-ku, Tokyo, 113-8656, Japan

E-mail: takahashi@i.u-tokyo.ac.jp

**
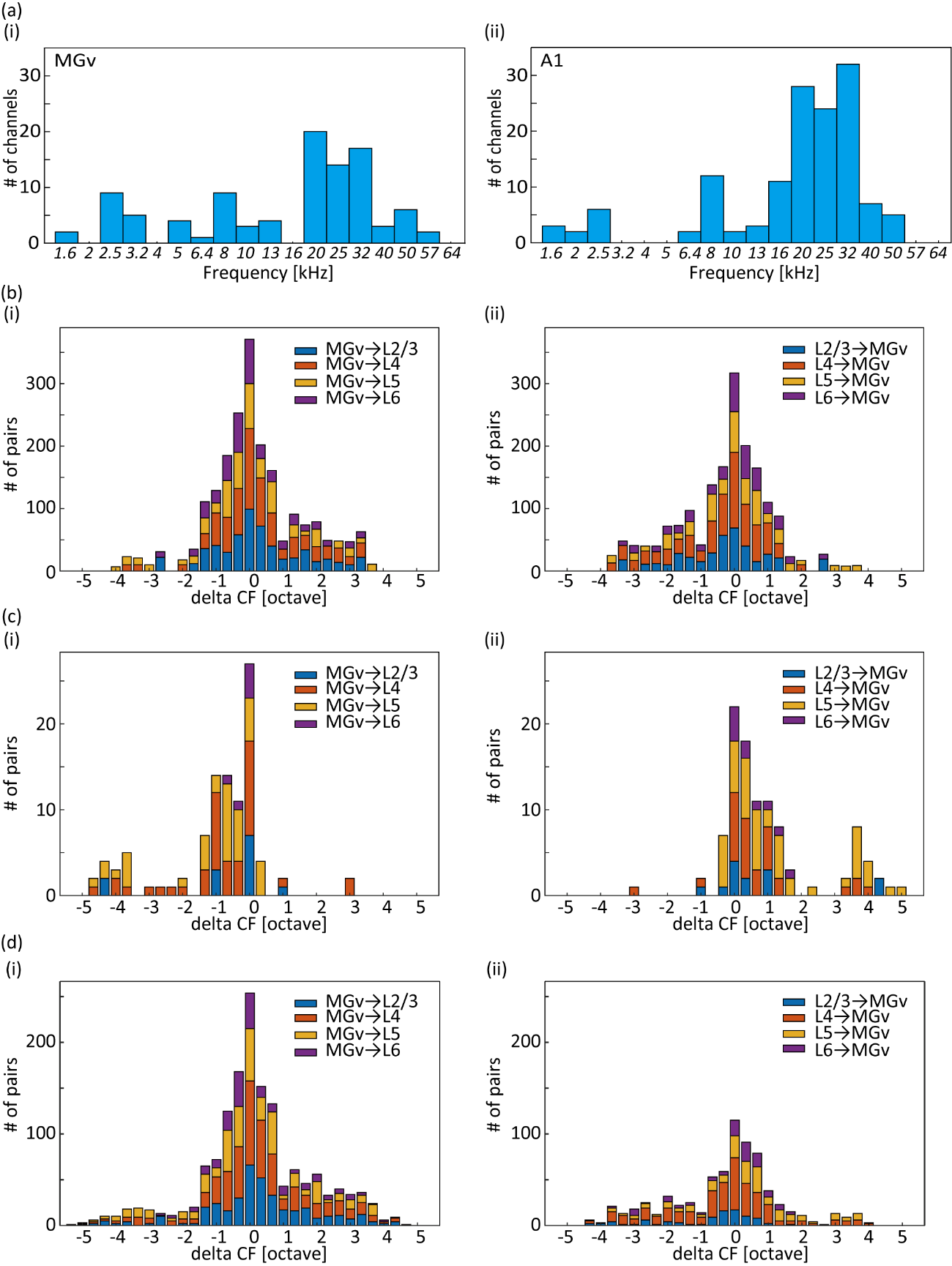
**

**Supplementary Fig. S1 Characteristic frequency (CF) of recording sites.**

(a) CF distribution in the MGv (i) and the A1 (ii) from the whole recording sites of test animals (N=4). (b) Delta-CF in long-window TEstim from MGv to A1 (i) and from A1 to MGv (ii). A1 sites were divided into layers: L2/3 (blue), L4 (orange), L5 (yellow) and L6 (purple). For a significant TE pair, the difference in octave between CFs at each site was defined as delta-CF. Positive delta-CF indicated that information transmitted from low to high CF sites. (c) Delta-CF in long-window TEspon. (d) Delta-CF in short-window TE.


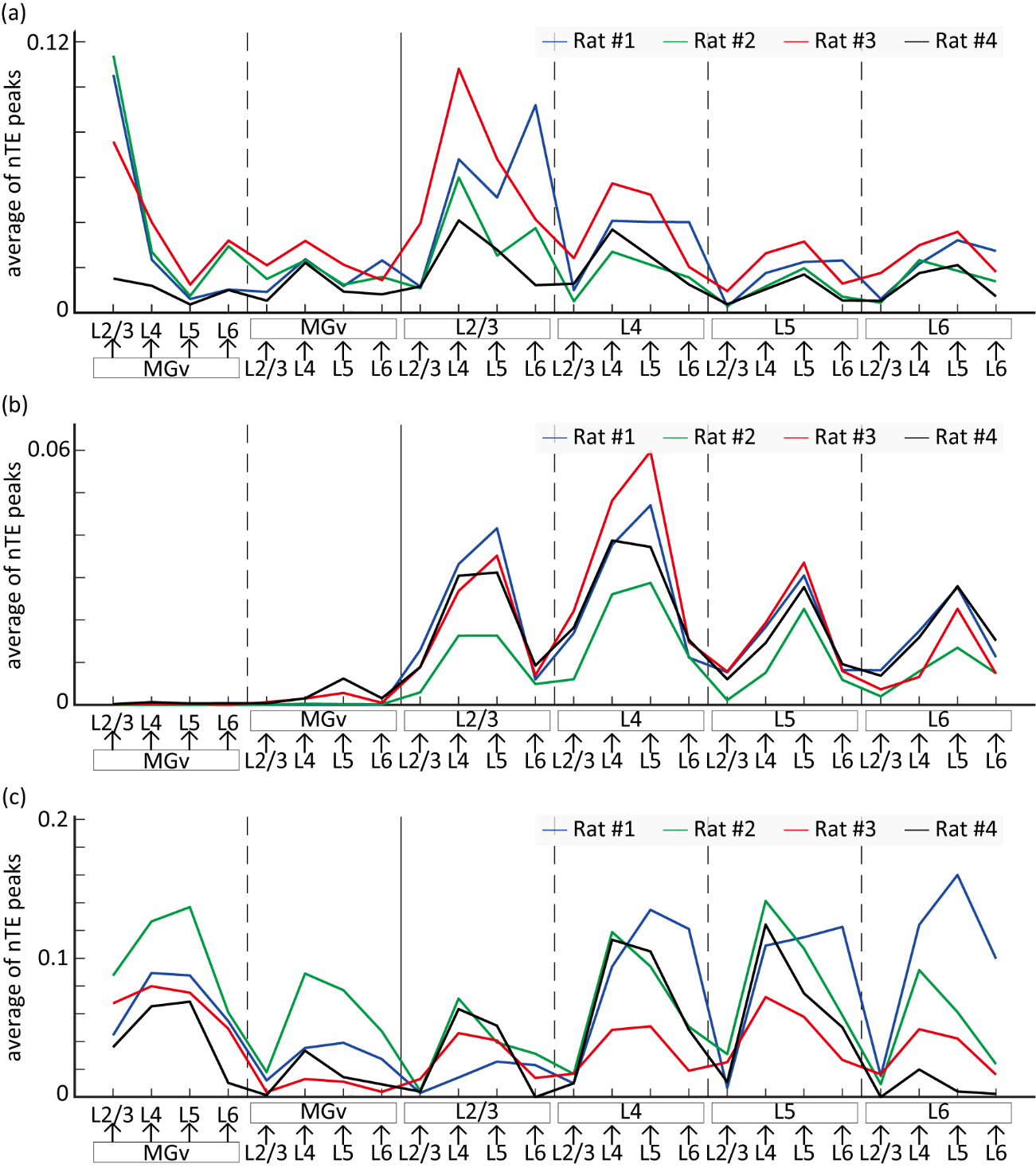


**Supplementary Fig. S2 Information flow and information transmission in individual animals.**

(a) Long-window TEstim. (b) Long-window TEspon. (c) Short-window TE. Graph patterns from individual animals (N=4) were shown as the average of nTE peaks in totally 24 pathways among cortical nodes (L2/3, L4, L5, and L6) and nodes in MGv; 4 thalamo-cortical pathways, 4 cortico-thalamic pathways, and 16 intracortical pathways. The correlation coefficients of these patterns across subjects were shown in solid lines: TEstim, 0.665 ± 0.185 (mean ± s.d.; t-test, p < 0.05 in 5 out of 6 pairs); TEspon, 0.954 ± 0.013 (P < 10^-11^ for all pairs); and short-window TE, 0.629 ± 0.148 (P < 0.05 for all test pairs).

**
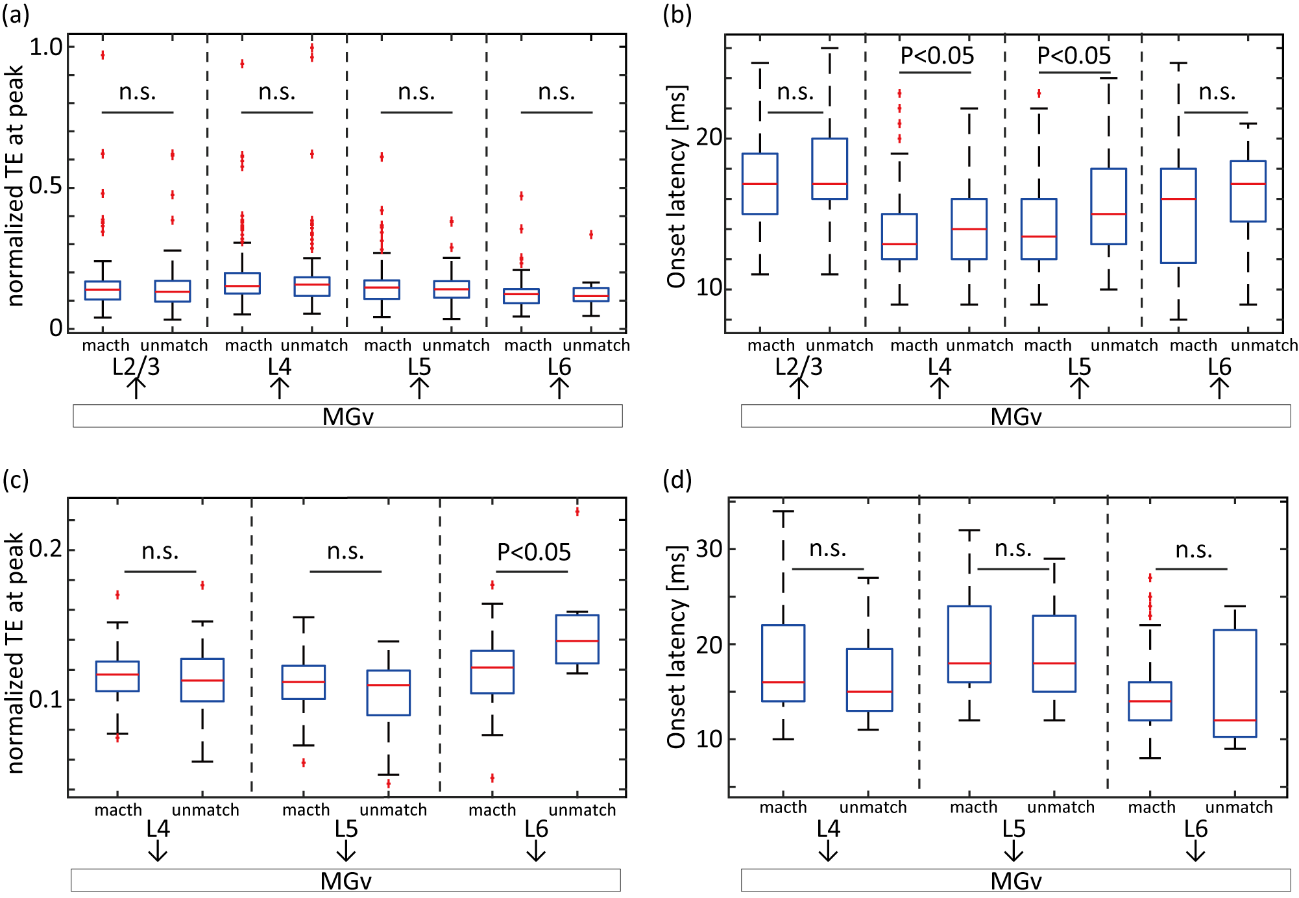
**

**Supplementary Fig. S3 Information transmission along a tonotopic axis in short-window TE.**

(a) Normalized TE (nTE) at peak from MGv to A1 was compared in pairs with matched CFs (delta CF ≤ 1/3 octave) and in pairs with unmatched CFs (delta CF > 1 octave). A1 sites were divided into layers (L2/3, L4, L5 and L6). (b) Onset latency of TE from MGv to A1. (c) nTE at peak from A1 to MGv. L2/3 was excluded from the analyses because of the small number of samples. (d) Onset latency of TE from A1 to MGv.
